# Supplementary material for: Content-rich biological network constructed by mining PubMed abstracts
Source: BMC Bioinformatics. 2004 Oct 8;5:147. doi: 10.1186/1471-2105-5-147 (PMC528731; doi:10.1186/1471-2105-5-147)
Supplement: Additional File 2 — The original results of the above study (non-essential files are deleted to keep the file size under the limit set by BMC bioinformatics). [file 1471-2105-5-147-S2.bz2 › chilibotAdditionalFile2/dip20/3ID9194558E4/html/TP53_BCL2.html]

 


 **TP53** and **BCL2** 
  
Found 77 abstracts in PubMed, retrieved 20.  
 

 What does Google say? 
 PDF only 
| .edu only 

---

**Interactive relationship** (e.g. stimulation, inhibition, etc)

**Non-interactive relationship** (e.g. studied together, co-existance, homology, etc.)

- No association between BCL6 status and expression of  **BCL2** , Ki 67 or  **TP53**  was found.  Ref: 11743658 Int J Oncol, 2002
- **TP53**  mutation, p21 and BCL 2  [ **BCL2** ]  expression were not associated with increased rates of progression and death.  Ref: 11720475 Br J Cancer, 2001
- Using molecular and immunohistochemical analyses we examined  **TP53**  and its downstream genes p21, BAX and BCL 2  [ **BCL2** ]  in ovarian tumour tissues and have evaluated the results in relation to clinico pathological parameters, clinical outcome and response to platinum based chemotherapy.  Ref: 11720475 Br J Cancer, 2001
- Another novel finding is that CR was negatively influenced by high BAX expression in all patients group P=0.047 and by  **BCL2**  expression in the  **TP53**  group P=0.05.  Ref: 12644821 Br J Cancer, 2003
- We investigated a series of 14 patients with PCNSL to determine structural alterations of the INK4a ARF, MDM2, and  **TP53**  genes, the status of bcl 2  [ **BCL2** ]  and bcl 6 protein expression, and the clinical course of the patients i.e. their survival time after diagnosis.  Ref: 11804283 J Neurooncol, 2001
- To elucidate the biochemical processes involved in the pathogenesis of B CLL and MCL, we analyzed the expression level of a set of genes that play central roles in apoptotic or cell proliferation pathways and of candidate genes from frequently altered genomic regions, namely ATM, BAX,  **BCL2** , CCND1, CCND3, CDK2, CDK4, CDKN1A, CDKN1B, E2F1, ETV5, MYC, RB1, SELL, TFDP2, TNFSF10, and  **TP53** .  Ref: 12036888 Blood, 2002
- To investigate the role of PML bodies in regulation of cell transformation and apoptosis related gene transcription, we employed the immuno FISH method to examine the relationship between PML bodies and the  **TP53**  and  **BCL2**  gene loci.  Ref: 12837275 Genomics, 2003
- PML bodies were found to localize specifically with the  **TP53**  locus in about 50% of Jurkat interphase nuclei, but never in proximity with the  **BCL2**  locus.  Ref: 12837275 Genomics, 2003
- Evaluation of clinical significance of  **TP53** , BCL 2  [ **BCL2** ] , BAX and MEK1 expression in 229 ovarian carcinomas treated with platinum based regimen.  Ref: 12644821 Br J Cancer, 2003
- However, initiation and continued progression toward cell death by apoptosis in cancer cells may be blocked by mutation of the tumor suppressor p53  [ **TP53** ]  or overexpression of members of the bcl 2  [ **BCL2** ]  family of proteins.  Ref: 12470246 Curr Med Chem, 2002
- We combined these approaches in a series of eight PCNSL cases, analyzing the chromosomal abnormalities using comparative genomic hybridization CGH, testing for Epstein Barr virus EBV involvement by in situ hybridization for EBER, assessing expression of p53, Bcl 2  [ **BCL2** ] , Bcl 6 and CD10 by means of immunohistochemistry, and screening for mutations of the  **TP53**  gene by DGGE.  Ref: 12802586 Virchows Arch< MedlineTA>Virchows Arch, 2002
- Reduced expression of BAX is associated with poor prognosis in patients with epithelial ovarian cancer a multifactorial analysis of  **TP53** , p21, BAX and BCL 2  [ **BCL2** ] .  Ref: 11720475 Br J Cancer, 2001
- Cytotoxicity because of substantial NO formation is established to initiate apoptosis, characterized by upregulation of the tumor suppressor p53  [ **TP53** ] , changes in the expression of pro and antiapoptotic Bcl 2  [ **BCL2** ]  family members, cytochrome c relocation, activation of caspases, and DNA fragmentation.  Ref: 12086396 J Environ Pathol Toxicol Oncol, 2002
